# Geospatial patterns of comorbidity prevalence among people with osteoarthritis in Alberta Canada

**DOI:** 10.1186/s12889-020-09599-0

**Published:** 2020-10-15

**Authors:** Xiaoxiao Liu, Rizwan Shahid, Alka B. Patel, Terrence McDonald, Stefania Bertazzon, Nigel Waters, Judy E. Seidel, Deborah A. Marshall

**Affiliations:** 1 Department of Community Health Science, Cumming School of Medicine, University of Calgary, 3280 Hospital Drive NW, HRIC Building, Room 3C58, Calgary, AB T2N 4Z6 Canada; 2 McCaig Bone and Joint Health Institute, University of Calgary, Calgary, Canada; 3 O’Brien Institute for Public Health, University of Calgary, Calgary, Canada; 4 Department of Geography, University of Calgary, Calgary, Canada; 5 Applied Research and Evaluation Services, Alberta Health Services, Calgary, Canada; 6 Department of Family Medicine, Cumming School of Medicine, University of Calgary, Calgary, Canada

**Keywords:** Osteoarthritis, Comorbidity, Spatial analysis, Rural, Disparities

## Abstract

**Background:**

Knowledge of geospatial pattern in comorbidities prevalence is critical to an understanding of the local health needs among people with osteoarthritis (OA). It provides valuable information for targeting optimal OA treatment and management at the local level. However, there is, at present, limited evidence about the geospatial pattern of comorbidity prevalence in Alberta, Canada.

**Methods:**

Five administrative health datasets were linked to identify OA cases and comorbidities using validated case definitions. We explored the geospatial pattern in comorbidity prevalence at two standard geographic areas levels defined by the Alberta Health Services: descriptive analysis at rural-urban continuum level; spatial analysis (global Moran’s I, hot spot analysis, cluster and outlier analysis) at the local geographic area (LGA) level. We compared area-level indicators in comorbidities hotspots to those in the rest of Alberta (non-hotspots).

**Results:**

Among 359,638 OA cases in 2013, approximately 60% of people resided in Metro and Urban areas, compared to 2% in Rural Remote areas. All comorbidity groups exhibited statistically significant spatial autocorrelation (hypertension: Moran’s I index 0.24, z score 4.61). Comorbidity hotspots, except depression, were located primarily in Rural and Rural Remote areas. Depression was more prevalent in Metro (Edmonton-Abbottsfield: 194 cases per 1000 population, 95%CI 192–195) and Urban LGAs (Lethbridge-North: 169, 95%CI 168–171) compared to Rural areas (Fox Creek: 65, 95%CI 63–68). Comorbidities hotspots included a higher percentage of First Nations or Inuit people. People with OA living in hotspots had lower socioeconomic status and less access to care compared to non-hotspots.

**Conclusions:**

The findings highlight notable rural-urban disparities in comorbidities prevalence among people with OA in Alberta, Canada. Our study provides valuable evidence for policy and decision makers to design programs that ensure patients with OA receive optimal health management tailored to their local needs and a reduction in current OA health disparities.

## Background

Osteoarthritis (OA) is the most common form of arthritis affecting 1 in 8 (13%) Canadians. By 2040, it is expected to be prevalent among 1 in 4 Canadians due to an aging population and increasing rates of obesity [1, 2]. The high prevalence of OA has a substantial impact on quality of life and health care costs to individuals and health care systems. Studies suggest that the quality of life among people with OA is 10–25% lower, and the annual health care costs per individual is two to three times higher, compared to the general population [3]. People with OA are more likely to have comorbid chronic conditions than the general population [4–6] which adds complexity to the patient management and routine clinical practice [7, 8]. However, most clinical practice guidelines are limited to addressing the management strategies when dealing with comorbidities coexisting with OA [9], which may result in poor quality of care due to the non-optimal health care provided for patients [10]. It is reported that the presence of comorbidities among people with OA resulted in increased physical disability compared with those without OA [11]. Literature from a wide range of countries using different study designs and sample sizes have reported comorbidities among people with OA [12, 13]. Among those frequently reported comorbidities, depression, chronic obstructive pulmonary disease (COPD) and hypertension were identified as the most prevalent conditions coexisting with OA in Alberta Canada, with almost half of OA cases having any combination of these three comorbidities [4].

Both the prevalence of comorbidity and associated management of these conditions have an inherent spatial nature due to the geographic variation of known health indicators [14]. A report of arthritis prevalence estimates suggested that arthritis prevalence, risk factors and management of arthritis across the US states varied substantially by geographic area [15]. Studies have examined the prevalence of arthritis in Canada at both provincial and regional level [8, 9], [16], and the prevalence of OA at local level [17]. OA had a substantially higher prevalence rate in Rural Remote and Rural areas than the Metro and Urban areas [17]. The Canadian Medical Association and Alberta Health Services (AHS) have a goal of achieving equitable access to care, with a focus on reducing health disparities for patients in rural and remote areas [18, 19]. However, there is no evidence regarding the geospatial pattern of comorbidity prevalence.

Alberta is Canada’s 4th largest by population and 6th largest by area [20], having a vast rural area in the northern half and the southwestern boundary. AHS is Canada’s first and largest province-wide, fully-integrated health system, the single health authority in Alberta being responsible for delivering better coordinated health care across the province. Like other Canadian provinces, people living in rural areas have less access to health care compared to their urban counterpart [14]. In this paper we aimed to identify local areas with significantly higher comorbidity rates by exploring the geospatial pattern of comorbidities prevalence at the local level in Alberta, Canada using spatial statistics. Our study will provide valuable evidence for policy and decision makers to design programs that will ensure patients with OA receive optimal management tailored to their local needs and a reduction in current OA health disparities.

## Methods

### OA prevalent cases in 2013

Our data sources were five Alberta Health (AH) administrative databases: Alberta Health Care Insurance Plan population registry (AHCIP), Physician Claims, Discharge Abstract Database (DAD), and Ambulatory Care Classification System (ACCS) /National Ambulatory Care Reporting System (NACRS) [4, 17, 22]. The AHCIP population registry captures individual level data (age, sex, postal code, death, etc.) on all persons who accessed health care services paid for by the provincial health care insurance plan. Each patient is assigned a unique patient identifier which serves to link datasets prior to deidentification. Members of the Armed Forces and the Royal Canadian Mounted Police, federal penitentiary inmates and Albertans who have opted out of the AHCIP are excluded [22]. Physician Claims captures outpatient fee-for-service billing data for publicly funded physician services. DAD records inpatient care data for all hospitalized patients. ACCS/NACRS captures ambulatory visit service utilization data for traditional hospital-based programs, community-based outpatient clinics and publicly funded hospital support services such as physiotherapy and occupational therapy [4].

Data from April 1994 through March 2013 were used to identify individuals with OA using the most current and validated case definition for administrative data: at least one OA hospitalization, or at least two OA physician visits or OA-related ambulatory care visits within 2 years, and none of the physicians or ambulatory care visits being on the same day [22, 23]. The OA-related records were identified as those with the first 3 digits 715 or M15 to M19 based on the ninth and tenth revisions of the International Classification of Diseases (ICD) codes, respectively [22]. The spatial analysis focused on the OA prevalent cases in 2013, which included 359,638 adult cases (≥18 years of age at diagnosis) who were identified as OA while residing in Alberta (1994–2013) and did not migrate out of the province or die between 1994 and 2013 fiscal years.

### Definition of comorbidity

The comorbidities included in this study were selected based on a scoping review of frequently reported comorbidities that co-occurred with OA, clinician consultation, and availability of validated algorithms using administrative data, which has been described in detail by Marshal et al. (2019) [4]. Eight commonly reported comorbidities among people with OA were identified in our study: hypertension, depression, chronic obstructive pulmonary disease (COPD), diabetes, myocardial infarction, cerebrovascular disease, congestive heart failure and peripheral vascular disease. People with specific comorbid condition were identified as those having 1 or 2 hospitalization or physician claims within 3 years on or prior to the diagnosis of OA according to their specific validated case definition [4]. Detailed ICD 9 and ICD 10 diagnostic codes used to identify each comorbidity are provided in an online supplementary appendix 1 [24–33]. We grouped individuals by the number of comorbidities that co-occurred with OA: no comorbidity; 1 comorbidity; 2 comorbidities; and 3+ comorbidities [4, 5, 34]. We also grouped individuals by type of comorbidity [1, 4]: OA with hypertension only; OA with depression only and OA with COPD only, as these three comorbidities are the most prevalent chronic conditions co-occurred with OA. The number of cases for diabetes, myocardial infarction, cerebrovascular disease, congestive heart failure and peripheral vascular disease were too low to run spatial statistics. We excluded these groups for analysis by type of comorbidities.

### Standard geographic areas

**Fig. 1:**
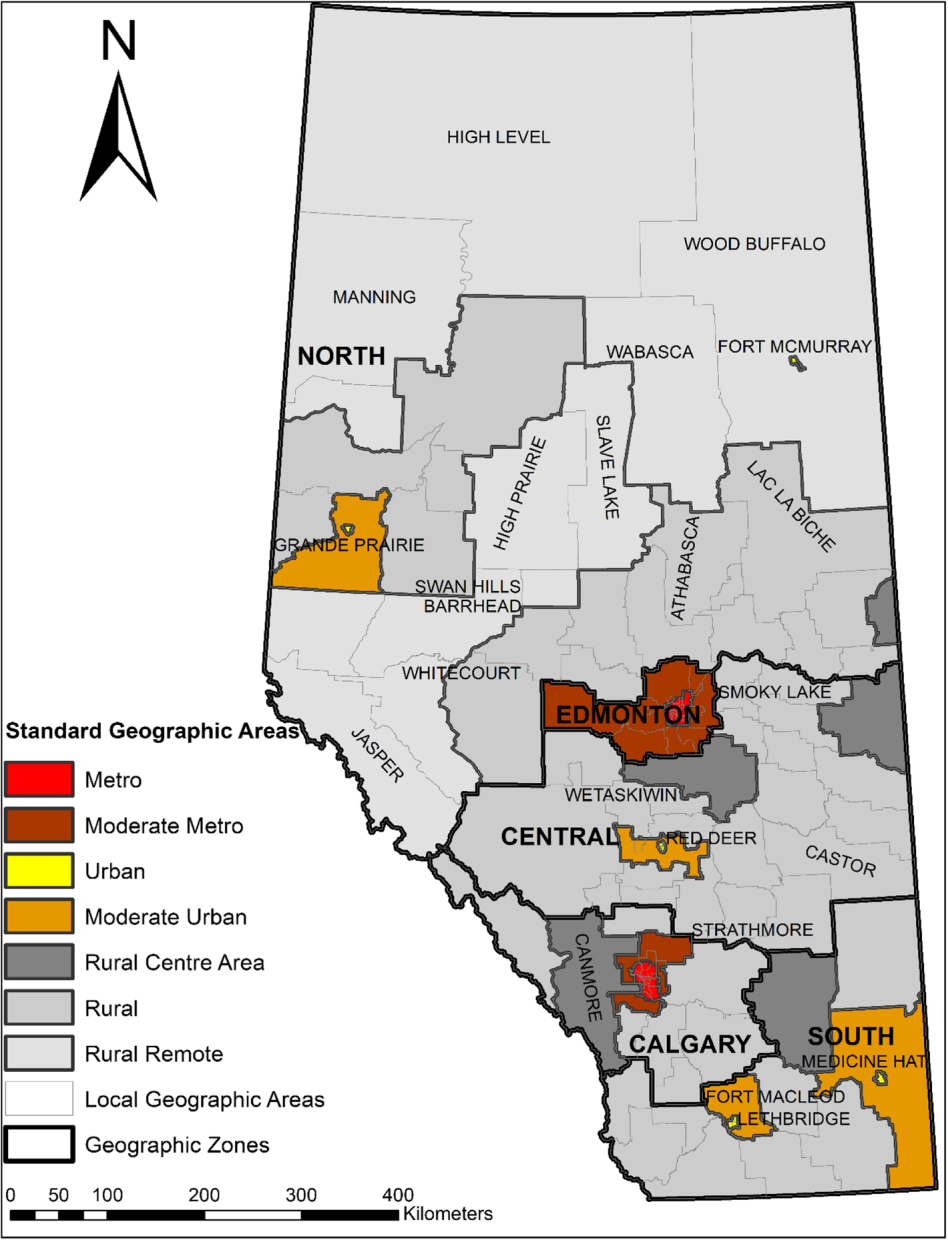
Standard geographic areas in Alberta. The authors created the map using ArcMap 10.8 through the University of Calgary Educational Site License

The standard geographic areas used in this analysis are existing units that were jointly created by Alberta Health (AH) and Alberta Health Services (AHS) for the purpose of planning and reporting of population health, health outcomes, and health services across Alberta [35] (Fig. 1). In addition to planning, LGAs are used regularly for research as they allow for findings to be applied into practice [36]. AHS Zones were formed for directing operational issues, including North Zone, Edmonton Zone, Central Zone, Calgary Zone and South Zone. The lowest geographic level is Local Geographic Area (LGA), which was developed for the purpose of providing detailed information particularly for health service planning. Across the province, 132 LGAs were created with populations varying from very small in rural area (as low as 1784) to large in metropolitan centres (up to 116,324), with a median population of 18,062 in 2011. The rural-urban continuum areas were created based on the aggregation of LGAs for the purpose of planning, monitoring, and comparing population health by rural-urban status. The province was stratified into seven distinct categories (Metro, Moderate Metro influence, Urban, Moderate Urban influence, Rural Centre, Rural, and Rural Remote) based on population density, distance from urban centres, and local knowledge of populations, industry type, municipalities, resources, and infrastructure [35].

### Descriptive analysis

We applied descriptive analysis to capture the characteristics of population in each comorbidity group. Counts and percentages of comorbidities among OA population were provided in Table 1. OA cases were grouped by sex and 7 age categories (18–35, 35–44, 45–54, 55–64, 65–74, 75–85, and > =85 years) at the rural urban continuum level. While at the LGA level, we grouped OA cases using broader age categories (18–44, 45–64, > = 65) to avoid small numbers of individuals in each stratum which would lead to unreliable rates. The crude rate per 1000 OA population was calculated as the number of people with comorbidities divided by the number of people with OA. Using the population registered in the AHCIP in 2013 as the standard population, we calculated the age-sex standardized prevalence rates with a direct standardization method [21]. Confidence intervals (CIs) of prevalence rates were calculated using a binomial approximation at 95% significance level [21]. The standardized rates ratio (SRR) was calculated as the rate of each geographic area divided by the rate in the rest of Alberta to avoid the effect of comparing heavily populated areas with the rate of Alberta as a whole. The 95% CIs of SRR were calculated with an approximation method to test if the difference between a given geographic area and the rest of Alberta was statistically significant [21]. Rates were calculated at both the rural-urban continuum area and the LGA level.

**Table 1:** Frequency and percentage of comorbidities among people with OA

Comorbidity Type	n	% (OA population 2013)	% (OA with Comorbidities)
*OA with 1 Comorbidity*	*120,936*	*33.6%*	*64.9%*
Hypertension (HTN)	46,871	13.0%	25.2%
Depression (DEP)	38,248	10.6%	20.5%
Chronic Obstructive Pulmonary Disease (COPD)	25,495	7.1%	13.7%
Diabetes (DIAB)	7794	2.2%	4.2%
Congestive Heart Failure (CHF)	1053	0.3%	0.6%
Peripheral Vascular Disease (PVD)	1014	0.3%	0.5%
Myocardial Infarction (MI)	323	0.1%	0.2%
Cerebrovascular Disease (CEVD)	138	0.0%	0.1%
*OA with 2 Comorbidities*	*47,909*	*13.3%*	*25.7%*
*OA with 3+ Comorbidities*	*17,505*	*4.9%*	*9.4%*
**OA with Comorbidities**	**186,350**	**51.8%**	
**OA with No Comorbidities**	**173,288**	**48.2%**	
**Total - OA in Alberta**	**359,638**	**100.0%**	

Note: n is the counts of population with listed comorbid conditions. % (OA population 2013) was calculated using OA population (*n* = 359,358) as denominator. % (OA with comorbidities) was calculated using population with at least 1 comorbidity at the diagnosis of OA (*n* = 186,350) as denominator

### Spatial analysis

LGAs was used for spatial analysis as they are the lowest geographic level that the AHS and AH use as official geographies. LGAs provide detailed spatial information as well as a sufficient number of cases for calculating accurate and reliable rates. Should the number of cases be lower than 20, estimates may be unreliable, with high variation due to small numbers [37, 38]. Alberta LGAs do not exhibit this problem, as over 95% of the 132 LGAs had a number of cases greater than 20 for all comorbidity groups.

The residential 6-digit postal code of each patient was obtained from the AHCIP population registry dataset, geocoded using Alberta Health Postal Code Translator File [39] and aggregated at the LGA level. Four postal codes with missing coordinates were excluded. Each postal code was assigned a pair of latitude and longitude coordinates, which was used to link postal code to a 2011 Census Dissemination Areas defined by Statistics Canada [40]. Dissemination Areas are the common building blocks used by the AHS and AH to aggregate the smallest areas into LGAs and define a rural-urban continuum [35].

Global Moran’s I was applied to test the presence of spatial autocorrelation [41]. Spatial autocorrelation is the degree of similarity or spatial dependence of comorbidities prevalence rates across LGAs as a function of their distance. In other words, global Moran’s I assesses whether LGAs with similar comorbidity rates tend to be located close together, far apart, or distributed randomly across the province. Moran’s I index ranges from − 1 to 1, with positive values suggesting spatial autocorrelation and negative values suggesting dispersion (i.e., similar rates located far from each other), and values close to 0 indicating a random distribution (i.e., the location of similar rates is independent of their distance). The null hypothesis was that the data followed a completely random distribution over space. The alternative hypothesis was that the observed pattern was more clustered or dispersed than what would be expected if the underlying spatial processes were random. The critical values of plus or minus 1.96 for Z scores and a *p* = 0.05 were applied to make decisions regarding accepting or rejecting the null hypothesis [42].

Global Moran’s I informs as to whether the comorbidity rates tend to be clustered (vs. randomly or regularly spaced), but does not tell us the location of the clusters. To locate LGAs with clustered comorbidities, we further applied hot spot analysis (based on the Getis-Ord Gi* statistic) [43, 44], as well as cluster and outlier analysis (based on local Moran’s I) to analyze the comorbidity distribution pattern. The two analytical techniques highlight the different aspects of a spatial pattern, and some research suggests they may be complementary [45, 46]. Hot spot analysis based on Getis Ord Gi* statistics identifies clusters of LGAs characterized by similar comorbidity rates [43, 44]. Within a predefined neighborhood, the statistic first calculates the sum of comorbidity rates at the LGA of interest and its neighbors within the defined neighborhood, then compares it proportionally to the sum of all LGAs. The critical Z score of 1.96 and a *p* = 0.05 were used to identify statically significant hotspots.

Cluster and Outlier Analysis was applied to examine spatial outliers surrounded by dissimilar values [43]. This analysis was used to calculate a local Moran’s I index for each LGA based on its similarity with neighboring LGAs within a defined neighborhood. Based on the computed z-score, *p*-value and local Moran’s I index, each LGA is classified by cluster/outlier type [46, 47]: a High-High (HH) cluster (a high rate surrounded by high rates); a Low-Low (LL) cluster (a low rate surrounded by low rates); a High-Low (HL) outlier (a high rate surrounded by low rates); a Low-High (LH) outlier (a low rate surrounded by high rates) and non-significant areas.

Essential to the spatial analysis discussed above is the definition of neighborhood, that is, a geographic area to which the spatial analysis is applied based on the hypothesis that each spatial unit [in our case each LGA] is more likely to interact with the other units in its neighborhood than with those outside [45, 48]. Unfortunately, we could not rely on literature or prior knowledge to conceptualize a proper neighborhood; therefore, we only hypothesized that LGAs located close by may exhibit greater similarity in OA comorbidity prevalence, compared to those further away, given their geographic proximity, rural-urban status, and similarity of risk factors and access to health care services. However, this basic hypothesis did not allow us to determine a rigid definition of the structure and size of the neighborhood.

Consequently, we assessed three different neighborhood types: 1) the first order queen polygon contiguity [48]; 2) the fixed distance band method [49], and 3) a specified minimum number of neighbors. The first order queen contiguity assumes that LGAs sharing a common boundary or vertex form a neighborhood. The 40 km distance band was defined based on the average distance of 37 km between LGA centroids and the 1st nearest neighbor, to ensure each LGA has at least 1 neighbor on average. To account for the varying size of LGAs, a minimum of 8 nearest neighbors was specified for both the queen contiguity and fixed distance methods to ensure that each neighborhood included at least 8 neighbours even for areas that would not meet this minimum requirement.

Spatial weights were assigned equally to the LGAs within the defined neighborhood [48], that is, no distance decay function was applied. This resulted in a binary specification, whereby all units inside a neighborhood are equally likely to interact with each other, whereas those outside are not. To account for the effect of the varying number of neighbors using the queen contiguity approach, row standardization was used for the spatial weights matrix calculation [45].

### Area-level indicators of hotspots versus non-hotspots

AH has developed reports on Community Profiles to assist with primary health care planning, offering an overview of current health status and future health needs of residents at LGA level. Using Community Profiles, we explored the characteristics of socioeconomic indicators and primary health care indicators in identified hotspots compared to those in the rest of Alberta (non-hotspots) (Table 2). The average number of indicators in hotspots and non-hotspots was compared by calculating the ratio between them. We applied Student’s *t-*test to test the statistical significance of difference in indicators between hotspots and non-hotspots.

**Table 2:** Indicators used for exploring the characteristics in hotspots versus non-hotspots

Indicators		Definition
Socio-Economic Indicators	**Aboriginal (%)**	Percent of population that is First Nations or Inuit
	**AvgIncome ($)**	Average Census Family Income ($)
	**University (%)**	Percent of population with university certificate, diploma or degree
Utilization indicators	**ACSC.Rate**	Ambulatory Care Sensitive Conditions-Age-Standardized Separation Rate (per 100,000 population)
Health Status Indicators	**COPD.Rate**	Chronic Obstructive Pulmonary Disease Prevalence Rate (per 100 population), 2010
	**Cmb3.Rate**	Age-Standardized Rate of People with Three or more Chronic Diseases (per 100 population), 2010

Descriptive analysis was conducted using R 3.6.1. Spatial analysis was conducted using ArcMap10.8. All the maps were created using ArcMap 10.8 through the University of Calgary Educational Site License.

## Results

Among 359,638 OA cases in 2013, 51.8% were identified having at least 1 of the 8 selected comorbidities (Table 1). The population with 1 comorbid condition accounted for one third of the total OA population (33.6%), which is 2.5 and 6.9 times as many as those with 2 comorbidities (13.3%) and those with 3+ comorbidities (4.9%), respectively. Among the population of OA with 1 comorbidity, hypertension is the most frequent condition (13%), followed by depression (10.6%) and COPD (7.1%). Approximately 60% of people with any comorbidities resided in the Metro and Urban areas, while the proportion of people residing in Remote Rural areas ranged from 2 to 4% among comorbidity groups (Table 3).

**Table 3:** Age-sex standardized rates of comorbidity among people with OA by rural-urban continuum

**a) Number of comorbidities (0–8)**													
**Geographic areas**	**OA cases**	**1 Comorbidity**				**2 Comorbidity**				**3+ Comorbidity**			
	**n**	**n**	**Crude Rate**	**Std.Rate**	**SRR**	**n**	**Crude Rate**	**Std.Rate**	**SRR**	**n**	**Crude Rate**	**Std.Rate**	**SRR**
Metro	176,502	59,780	338.7	302.6 [302.4–302.8]	0.98 [0.98–0.98]	23,624	133.8	88.8 [88.8–88.9]	0.94 [0.94–0.94]	8384	47.5	22.2 [22.2–22.3]	0.88 [0.87–0.88]
Moderate Metro	48,781	16,412	336.4	299.1 [298.7–299.4]	0.97 [0.97–0.98]	5925	121.5	81.2 [81.1–81.4]	0.87 [0.87–0.87]	2033	41.7	20.8 [20.8–20.9]	0.86 [0.86–0.86]
Urban	32,038	10,672	333.1	314.4 [313.9–314.9]	0.96 [0.96–0.96]	4237	132.2	86.1 [85.9–86.3]	0.93 [0.93–0.94]	1713	53.5	26.9 [26.8–27.0]	1.15 [1.15–1.16]
Moderate Urban	7389	2415	326.8	293.2 [292.4–294.0]	1.00 [1.00–1.00]	915	123.8	91.4 [91.0–91.9]	1.00 [0.99–1.00]	327	44.3	20.0 [19.9–20.1]	0.84 [0.84–0.84]
Rural Centre Area	16,399	5562	339.2	330.1 [329.4–330.9]	1.08 [1.08–1.09]	2380	145.1	107.8 [107.4–108.2]	1.19 [1.18–1.19]	938	57.2	30.3 [30.1–30.4]	1.29 [1.28–1.30]
Rural	70,408	23,303	331.0	306.1 [305.7–306.4]	1.13 [1.13–1.13]	9550	135.6	101.2 [101.1–101.4]	1.13 [1.13–1.13]	3615	51.3	27.6 [27.6–27.7]	1.20 [1.20–1.20]
Rural Remote	8121	2792	343.8	344.0 [343.0–345.0]	1.03 [1.03–1.03]	1278	157.4	124.9 [124.4–125.4]	1.38 [1.37–1.39]	495	61.0	27.3 [27.1–27.5]	1.15 [1.15–1.16]
**Alberta**	**359,638**	**120,936**	**336.3**	**305.6 [305.5–305.7]**		**47,909**	**133.2**	**91.5 [91.4–91.5]**		**17,505**	**48.7**	**23.7 [23.7–23.7]**	
**b) Selected single comorbidities**													
**Geographic areas**	**OA cases**	**HTN Only**				**DEP Only**				**COPD Only**			
	**n**	**n**	**Crude Rate**	**Std.Rate**	**SRR**	**n**	**Crude Rate**	**Std.Rate**	**SRR**	**n**	**Crude Rate**	**Std.Rate**	**SRR**
Metro	176,502	23,411	132.6	55.7 [55.7–55.8]	0.94 [0.94–0.94]	19,533	110.7	147.1 [146.9–147.2]	1.07 [1.07–1.07]	11,760	66.6	82.1 [82.0–82.2]	0.86 [0.86–0.86]
Moderate Metro	48,781	6335	129.9	60.9 [60.9–61.0]	1.07 [1.07–1.07]	5462	112.0	143.1 [142.9–143.4]	1.01 [1.00–1.01]	3371	69.1	79.4 [79.2–79.5]	0.88 [0.88–0.88]
Urban	32,038	3957	123.5	52.0 [51.8–52.1]	0.90 [0.89–0.90]	3537	110.4	146.8 [146.4–147.1]	1.03 [1.03–1.04]	2215	69.1	99.5 [99.2–99.8]	1.13 [1.13–1.14]
Moderate Urban	7389	794	107.5	45.4 [45.2–45.5]	0.79 [0.79–0.79]	774	104.8	132.2 [131.6–132.7]	0.93 [0.92–0.93]	640	86.6	100.4 [99.9–100.9]	1.13 [1.13–1.14]
Rural Centre Area	16,399	2212	134.9	61.6 [61.4–61.7]	1.08 [1.08–1.08]	1653	100.8	155.0 [154.4–155.5]	1.09 [1.09–1.10]	1220	74.4	96.3 [95.8–96.7]	1.09 [1.08–1.09]
Rural	70,408	9354	132.9	66.1 [66.1–66.2]	1.19 [1.18–1.19]	6524	92.7	125.9 [125.7–126.1]	0.86 [0.86–0.87]	5298	75.2	95.4 [95.2–95.6]	1.09 [1.09–1.10]
Rural Remote	8121	808	99.5	42.7 [42.5–42.8]	0.74 [0.74–0.74]	765	94.2	131.3 [130.6–132.0]	0.92 [0.91–0.92]	991	122.0	156.3 [155.5–157.0]	1.80 [1.79–1.81]
**Alberta**	**359,638**	**46,871**	**130.3**	**57.3 [57.3–57.4]**		**38,248**	**106.4**	**142.3 [142.2–142.4]**		**25,495**	**70.9**	**88.8 [88.7–88.9]**	

Note: SRR is the ratio comparing the rate of geographic area to the rest of Alberta. Std. Rate denotes the age-sex standardized rates

### Age-sex standardized rate by rural-urban continuum

The age-sex standardized prevalence of comorbidity varied across the rural-urban continuum (Table 3). People with 1, 2 and 3+ of the 8 comorbidities presented at the diagnosis of OA were observed to be most prevalent in Rural Remote and Rural Centre areas and least prevalent in Moderate Urban and Moderate Metro areas respectively. By type of comorbid conditions, the prevalence of COPD among people with OA ranged from the highest of 156 cases per 1000 OA population in Rural Remote areas (SRR 1.80, 95%CI 1.79–1.81) to the lowest of 82.1 (SRR 0.86, 95%CI 0.86–0.86) and 79.4 (SRR 0.88, 95%CI 0.88–0.88) cases in Metro and Moderate Metro areas respectively. However, the prevalence pattern of depression was different; it was most prevalent in Rural Centre areas (155 cases, SRR 1.09, 95%CI 1.09–1.10) and Metro (147 cases, SRR 1.07, 95%CI 1.07–1.07), and least prevalent in Rural (126 cases, SRR 0.86, 95%CI 0.86–0.87) and Rural Remote areas (131 cases, SRR 0.92, 95%CI 0.91–0.92).

### Spatial analysis at the LGA level

**Fig. 2:**
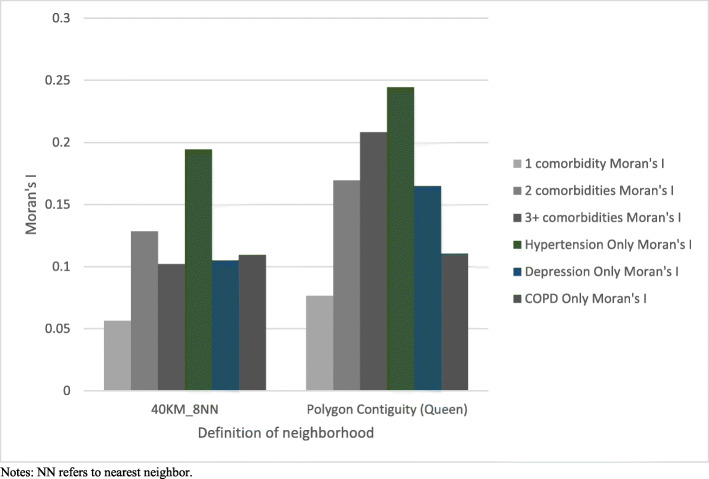
Global Moran’s I index by conceptualization of spatial relationships and by comorbidity group

**Fig. 3:**
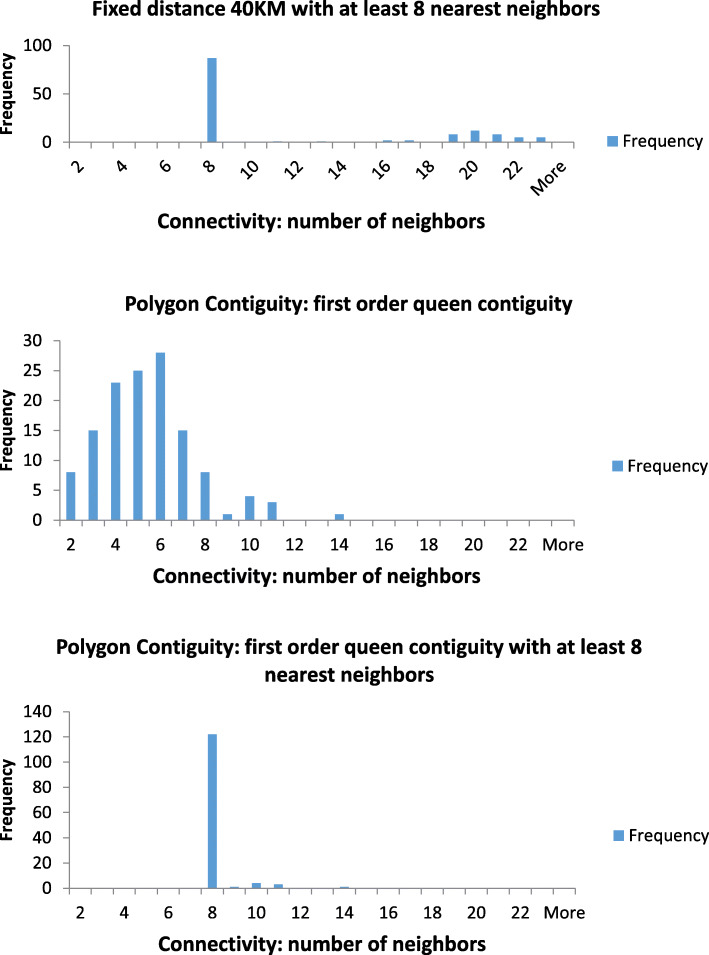
Connectivity histograms of three weight matrices

Among the 132 LGAs in Alberta, the smallest LGA was located in the Metro area (7 km^2^) and the largest in the Rural Remote area (99,994 km^2^) (Appendix 2). The average size of LGAs ranged from 50 km^2^ in Metro to 26,742 km^2^ in Rural Remote areas. As shown in Fig. 2, Global Moran’s I index for most comorbidity groups was higher using a spatial weights matrix based on queen contiguity (Z score: 3.95 for 3+ comorbidities), compared to the spatial weights matrix based on fixed distance (Z score: 2.65 for 3+ comorbidities). The first order queen contiguity produced 5.41 neighbors (Range: 1 to 14) on average with relatively low variance (Fig. 3). Conversely, the fixed distance band (40 km) method with the constraint of a minimum of eight neighbors yielded marked variability in the number of nearest neighbors. Comparing the three matrices, we preferred the unconstrained first order queen contiguity one, due to its low percentage of connectivity and nearly normal distribution of neighbor counts. The distribution of hypertension among people with OA exhibited statistically significant spatial autocorrelation, with a Moran’s I index of 0.24 (Z score = 4.61, *p* = 0.001).

We ran the Getis-Ord Gi* hot spots analysis using both the constrained and unconstrained first order queen contiguity spatial weight matrices. As shown in Appendix 3, though the identified hot spots using the two approaches mostly overlapped, the constrained matrix identified a broader set of hot spots in the South, where neighborhoods were larger. In order to limit such differences, as well as the impact of local specificities, we chose the unconstrained approach for the following analysis.

#### Hotspots and outliers

**Fig. 4:**
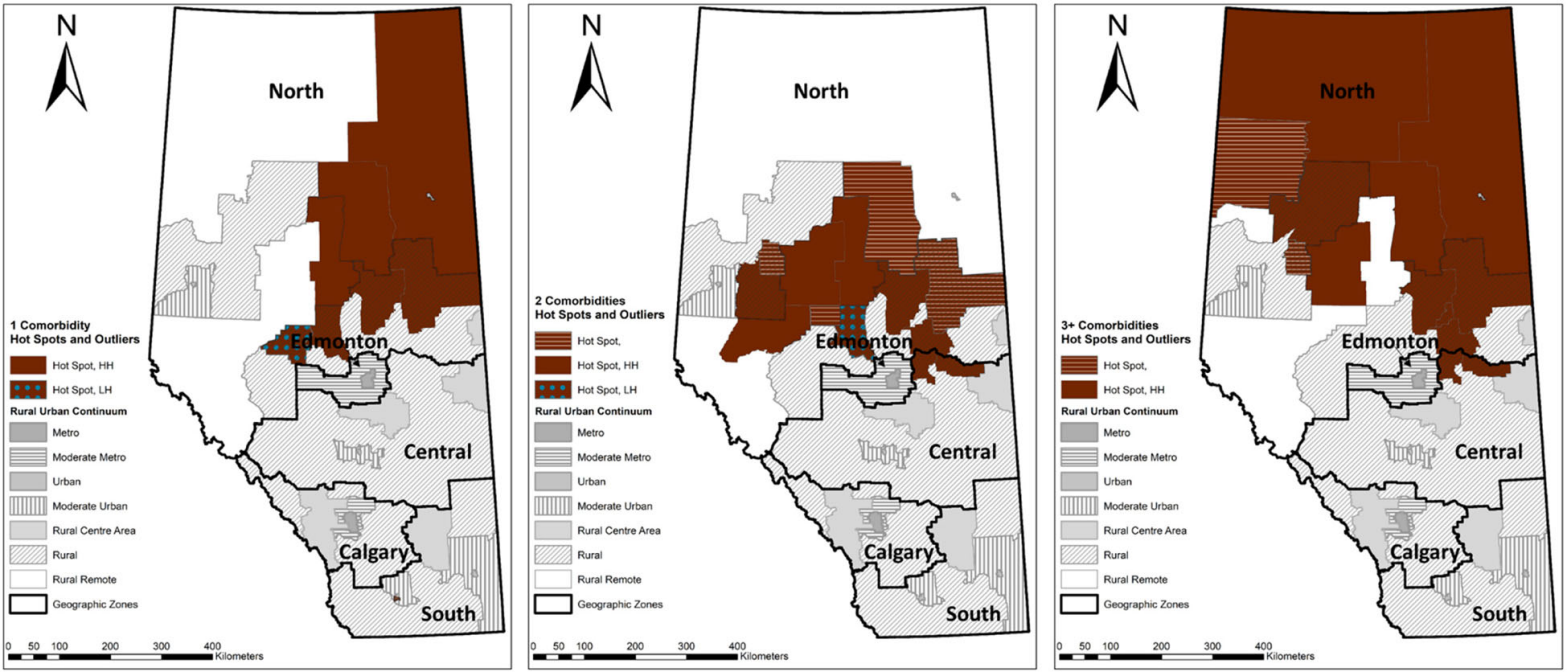
Hot spots and outliers for people with OA and 1 comorbidity (left), 2 comorbidities (middle), and 3+ comorbidities (right). The authors created these maps using ArcMap 10.8 through the University of Calgary Educational Site License

The number of hotspots identified for each comorbidity group ranged from 6 (depression) to 13 (COPD, 2 comorbidities, 3+ comorbidities). The prevalence of 1 comorbidity had 10 hotspots (Fig. 4), primarily located in the Rural and Rural Remote areas in the North Zone including Wabasca (409 cases per 1000 OA population) and Wood Buffalo (395). We also identified Whitecourt (310) as a low-high outlier with low value surrounded by high prevalence rate in its neighboring LGAs.

**Fig. 5:**
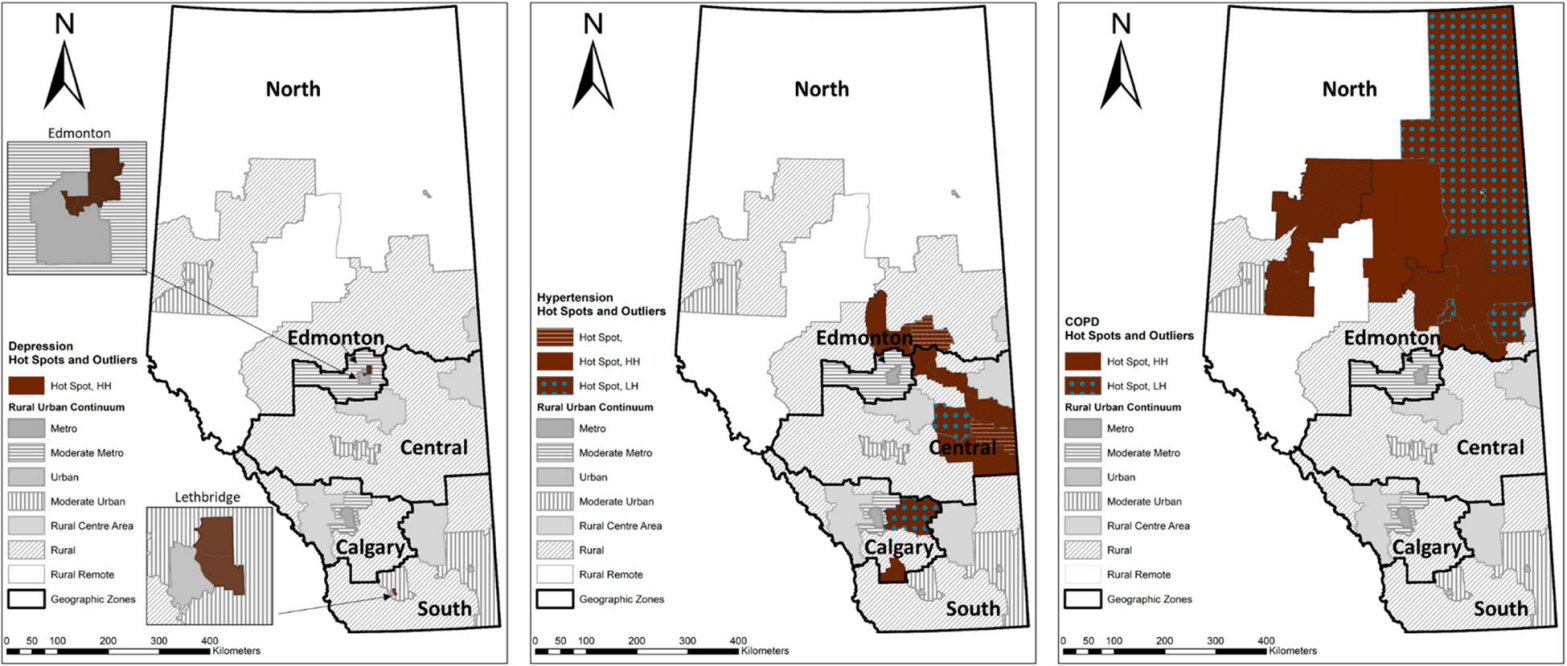
Hot spots and outliers for people with OA and depression (left), hypertension (middle), and COPD (right). The authors created these maps using ArcMap 10.8 through the University of Calgary Educational Site License

The prevalence of 2 comorbidities and 3 + comorbidities displayed a similar pattern as that of 1 comorbidity. However, people with 2 comorbidities were more prevalent in the northwest Rural Remote areas including Wabasca (216) and Swan Hills (212), while those with 3+ comorbidities were more prevalent in the far north Rural Remote areas including Manning (71), Wabasca (56), and High Level (44). As shown in Fig. 5, COPD had a similar pattern to the distribution of OA with 1 comorbidity, with clustered high prevalence rates in the north Rural Remote including Wabasca (240) and Slave Lake (111), and the north Rural areas including Athabasca (171) and Lac La Biche (162). Wood Buffalo was a low-high outlier having a prevalence rate of 65 cases, significantly lower than its neighbors: Wabasca (240) and Lac La Biche (162).

OA with hypertension displayed a different pattern from COPD (Fig. 5). All the 10 identified hotspots for hypertension were located in the Rural areas in the Central and Calgary zone including Provost (131) and Smoky Lake (113). Flagstaff were low-high outliers with hypertension prevalence rate of 57 cases surrounded by Wainwright (98) and Castor (90). The prevalence of depression was identified in 6 hotspots that were all located in Metro and Urban areas: Edmonton-Abbottsfield (194), Edmonton-Eastwood (193), Edmonton-NE (164), Edmonton-Woodcroft East (169), Lethbridge-North (169) and Lethbridge-South (147).

#### Area-level indicators of hotspots versus non-hotspots

The proportion of First Nations or Inuit population in the identified hotspots was almost 5 times as high as those LGAs identified as non-hotspots (1 comorbidity: 19.1% in hotspots vs. 4.6% in non-hotspots; 2 comorbidities: 16.0% in hotspots vs. 4.0% in non-hotspots; COPD: 22.0% in hotspots vs. 4.0% in non-hotspots) (Table 4). The average family income in hotspots was 10 to 20% lower than the non-hotspots (1 comorbidity: $77,907 in hotspots vs. $87,233 in non-hotspots; COPD: $77,336 in hotspots vs. $87,447$ in non-hotspots). The proportion of population with a university degree ranged from 11% in hotspots of 2 comorbidities to 16% in hot spots of those with 1 comorbidity among people with OA, which was 20 to 40% percent lower than those non-hotspots. The prevalence of COPD and 3+ comorbidities among general population was higher in hotspots as compared to non-hotspots. The differences between indicators in hotspots and non-hotspots were statistically significant for OA cases with 2 comorbidities, 3+ comorbidities, hypertension and COPD.

**Table 4:** Average indicators in hotspots versus non-hotspots

Comorbidity	Hotspots	Std.Rate	Aboriginal (%)	AvgIncome ($)	University (%)	ACSC.Rate	COPD.Rate	Cmb3.Rate
1 Comorbidity	Hotspots	351	19.1	77,904	16.0	1126.2	3.3	3.2
	Non Hotspots	310	4.6	87,233	20.0	626.4	2.1	2.4
	Ratio	1.1**	4.2	0.9	0.8	1.8**	1.6*	1.3*
2 Comorbidities	Hotspots	141	16.0	71,919	11.0	1164.2	3.8	3.6
	Non Hotspots	96	4.0	88,129	20.0	609.6	2.0	2.3
	Ratio	1.5**	3.6*	0.8**	0.6**	1.9**	1.9**	1.5**
3+ Comorbidities	Hotspots	43	20.0	70,529	11.0	1304.7	3.5	3.4
	Non Hotspots	27	4.0	88,283	20.0	594.2	2.0	2.4
	Ratio	1.6**	4.7**	0.8**	0.6**	2.2**	1.7**	1.5**
Hypertension	Hotspots	91	3.0	75,271	12.0	818.6	2.6	2.7
	Non Hotspots	67	6.0	87,451	20.0	651.6	2.1	2.4
	Ratio	1.4**	0.5	0.9**	0.6**	1.3**	1.2*	1.1*
Depression	Hotspots	173	6.0	69,132	21.0	541.4	2.3	2.7
	Non Hotspots	135	6.0	87,355	19.0	670.1	2.2	2.4
	Ratio	1.3**	1.0	0.8**	1.1	0.8**	1.1	1.1
COPD	Hotspots	124	22.0	77,336	12.0	1266.1	3.7	3.4
	Non Hotspots	84	4.0	87,447	20.0	604.0	2.0	2.4
	Ratio	1.5**	5.6**	0.9**	0.6**	2.1**	1.8**	1.5**

Note: ** denotes *p* < = 0.05; * denotes *p* < = 0.10

## Discussion

This paper examined the geospatial pattern of comorbidities prevalence among people with OA at the local level along the rural-urban continuum in Alberta. Our hot spot analysis showed that the prevalence rates of comorbidities, except for depression and hypertension, were significantly higher than average in the LGAs located primarily in the north Rural and Rural Remote areas. Depression on its own, was more prevalent in Metro and Urban areas compared to Rural areas. These findings may fill a gap in knowledge regarding the geospatial pattern in the prevalence of comorbidities among people with OA. The results highlight important rural-urban disparities regarding the local health needs and access to health care. They provide important information for the development of patient-centered care, ensuring appropriate management recommendations for health care programme and delivery [4].

The variation observed in our study may be partially explained by the spatial distribution of risk factors such as obesity, prior trauma, physical activity levels, ethnicity, family income and education [50]. Indeed, the Alberta Government reported that the percentage of obese people in the North Zone in 2013 was 27.4%, the highest percentage among all the five zones and much higher than the provincial percentage of 19.3%. In addition, a higher proportion of inactive people was reported in the North Zone compared to the province (43.8% vs. 42.6% Alberta) [51]. Overall, the North Zone had a higher prevalence of comorbidities among people with OA, a higher prevalence of obese populations and larger populations that were inactive. These two factors have been shown to be correlated with both early onset and increased disease progression in OA [50, 52].

The identified hotspots had a substantially higher percentage of First Nations or Inuit persons, and a lower socioeconomic status compared to non-hotspots, which may explain some of the geospatial pattern we observed. Studies have suggested that certain ethnicities have higher rates of OA - this includes First Nations in Canada [53, 54]. With regard to the socioeconomic factors, recent research has broadened the view of disease causation to include health risks that are associated with both individual socioeconomic circumstances such as education, income and occupation, and the contextual socioeconomic environment of one’s neighborhood [55]. The significant associations between OA outcomes and socioeconomic status have been reported in a number of studies, even after adjustment for the risk factors of age, race, BMI, knee injury and occupation [56, 57].

Our study identified a rural-urban disparity in the prevalence of comorbidities among people with OA. We found that Rural and Rural Remote areas have a high prevalence of comorbidities such as COPD among people with OA, which is in agreement with current literature [4]. These findings highlight a common issue for Canadian provincial health systems, namely that a rural-urban disparity exists in health outcomes and health care access [58]. It has been commonly reported that rural Canadians have higher health care needs but less access to health care [14, 21]. By comparing the experience of rural and urban dwelling seniors when seeking health services for OA, it has been shown that although rural and urban patients have shared some common experiences, rural patients reported a unique experience of having difficulty obtaining appointments and maintaining a general practitioner over the long term [59]. In Alberta, similar disparities exist. The prevalence of people with 3+ chronic diseases and people with COPD was observed to be most common in the North Rural and Rural Remote areas. These local areas were commonly identified as hotspots for comorbidities among people with OA, suggesting low health outcomes and high health care needs in the North Rural and Rural Remote areas. In addition, the ambulatory care sensitive conditions separation rates per 100,000 population [60], a valid proxy indicator for the robustness of a primary care system, was highest (1029.3) in the North Zone, especially in the Rural (939.6) and Rural Remote areas (1302.5), compared to the provincial level of 664.2. The disproportionately high rates reflected problems in obtaining access to appropriate primary care in the North Rural and Rural Remote areas. The results described here provide a better understanding of these geographic differences and may further inform planning of health care services. The Canadian Medical Association and AHS have a goal to achieve equitable access to care, with a focus on reducing health disparities for patients in rural and remote areas [18, 19]. In Alberta, community-based health and rehabilitation services continues to be promoted. This is complemented by over 40 primary care network organizations whose mandate is to meet the needs of the local patient populations as identified by area member Family Physicians. Our findings provide evidence for the development of patient-centered and coordinated health care that is responsive to local need, which may potentially reduce rural-urban disparities and achieve equitable access to care.

Unlike COPD, the prevalence of depression after age-sex standardization presented a pattern with a higher prevalence rate in Metro and Urban areas, compared to the Rural areas. This pattern is consistent with a number of reviews generally showing higher overall rates for mental disorders, more specifically, depression in urban areas [61, 62]. Researchers calculated the pooled total prevalence rates for psychiatric disorders, mood disorders and anxiety disorders respectively, which were found to have statistically significant associations with urbanization [63]. Romans et al. (2011) compared the geographic variability of rates of depression using the 2002 Canadian Community Health Survey, demonstrating that people living in the most rural environment have a low prevalence rate of depression (odds ratio = 0.76, 95%CI 0.59–0.98) [64].

An understanding of local areas with significantly higher prevalence of comorbidities provides valuable information to both AHS and Primary Care Networks, which are embedded throughout the province working together to address the health needs of local populations. Community-based physical activity interventions and the intervention of self-management education have resulted in significant reductions in pain and physical function associated with OA [65, 66]. However, the prevalence of comorbidities might complicate the management of OA and increase the negative outcomes. The presence of concurrent medical conditions is often a barrier to participating optimally in exercise programs. It is common in clinical practice to exclude patients with comorbidities from exercise therapy. Even if accepted, the exercise intensity tends to be lowered to a level that is less effective at OA management but without aggravating symptoms of the comorbid disease [67]. For example, the co-occurrence of COPD and OA may adversely affect management program such as pulmonary rehabilitation (PR) which aim to improve exercise capacity and health-related quality of life. However, there are no formal guidelines on the assessment and management of those with comorbidities [68]. To move PR programs closer to being patient rather than disease focused, it has been suggested that aquatic exercise is an effective substitute to land-based exercise for those with COPD and lower limb comorbidities [69]. The presence of comorbidities may influence the prescription of pharmacological therapy in patients affected by OA. Health care providers must be cautious about the drug interactions and adverse side effects when treating OA, pain and comorbid conditions holistically. Non-steroidal anti-inflammatory medications, the most common medications for pain management, are expected to be associated with various side effects that include gastrointestinal, renal and cardiovascular implications [70]. For patients with coexisting COPD and OA, the prescription of neutrophil elastase inhibitors, already used in the management of COPD, has been shown to have protective and reparative effects on joint inflammation [69]. Without knowledge of comorbidities at the local level, the management of OA may be counter-productive to improving care for people with multiple chronic conditions. Given the geospatial pattern in the prevalence of comorbidities, it is important to develop OA management based on local needs. Current clinical practice guidelines for OA management do not include recommendations regarding mental health management. As depression is more prevalent in Metro and Urban areas, educating physicians about timely identification of psychological factors may be helpful to improve outcomes. While in the Rural and Rural Remote areas with high prevalence of COPD and multimorbidites, comorbidity-adapted exercises program may be developed to improve function for patients with OA. Localized self-management programs may be targeted to hot spots to educate the public how to minimize the symptoms of OA and prevalent comorbidities. Patient-centered and coordinated care has been recommended in clinical practice guidelines that aim to improve the quality of care by focusing on the patient as a whole rather than on a single disease [71]. In addition to the Patient Medical Home that focuses and promotes team based care, incorporating a physiotherapist as the first-point of contact in primary care might be beneficial [72–75].

### Strength and limitations

The identified hot spots provide evidence-based information for a better understanding of the local clinical context for managing OA patients and a better targeting of appropriate OA treatment and management that is responsive to local needs. The OA prevalent cases were identified using long-term longitudinal data from 1994 to 2013, which is critical to estimate OA prevalence as research suggests approximately 15 years of longitudinal data is required to reach plateau [22]. Using administrative data from the province of Alberta, we captured a wider range of comorbidities that are clinically relevant to the management of people with OA.

A limitation of our study is that case identification using administrative data may result in underestimating the prevalence of OA and comorbidities. OA is likely underdiagnosed in primary care due to a large group of people with symptomatic OA not seeking health care, which may be explained by non-optimal management of OA in primary care and self-coping with non-prescription medications [76, 77]. It is quite common that OA-related pain and disability is perceived by patients as a part of normal aging, which may lead to patients having a high acceptance of symptoms and not seeking treatment. The underdiagnosis of OA may be greater in rural and rural remote areas where people experienced less access to care [14]. As the extent of such bias to different estimates of OA across the rural urban continuum is unknown, we have to be cautious about the interpretation of results.

The exclusions of those who died or moved out of Alberta for the OA estimates were consistent with the Alberta population estimates based on the AHCIP registry population, which captured people covered by the Alberta Health Care Insurance Plan. Those who died or migrated out of Alberta in the given year were no longer eligible for the health care insurance plan. As there was no exact number regarding the death and out-of-province population among people with OA, this study could not estimate the extent of bias to the OA prevalence estimates. However, we obtained estimates of death and migration population in 2012/2013 in the Alberta general population. The number of net immigrants (45,718) outnumbered the deaths (22,175), even without considering international immigrants and temporary foreign workers [78, 79]. Hence, we assumed that the bias caused by the exclusion criteria to the OA estimates might be minor. In addition, our study captured the levels of comorbidity among people with OA at the time of OA diagnosis only. New cases of comorbidity diagnosed after the OA diagnosis were not identified [4].

Alberta is unique in its integrated health care model which is used by the provincial government to administer and deliver public health care to all Albertans. Recently, other Canadian provinces have taken steps to better integrate their health care system. For example, Saskatchewan consolidated the province’s 12 health regions into one provincial health authority as of December 4, 2017. Ontario clustered 14 Local Health Integration Networks into five interim and transitional geographic regions in November 2019. This study of Alberta will benefit other regions by providing a useful example. Further, even with different health care models and different definitions of rural-urban geographic areas among provinces, rural-urban disparities in disease prevalence and access to health care have been a common issue in Canada. The specifics of our methodology may be different from other regions and countries, but the analysis of patterns may be conducted in a similar manner [80–82].

The edge effect problem is also a limitation. This leads to LGAs at the provincial boundaries having fewer neighbors in specific directions when compared to those located in the center of the province. We applied row standardization to mitigate this issue, a typical way to account for this asymmetry in the count of neighbors [45]. Though the edge effect could be study area dependent, a study in France stated that their spatial analysis results such as Moran’s I index and local indicators of spatial autocorrelation were not greatly impacted by the edge effect [83]. Further, the hot spots analysis based on Gi* statistics identified areas with clustered higher vales within the context of neighboring observations. But it could not determine which hotspots should have a higher priority than others for remedial activity. Similarly our study could not use the hot spot analysis as the sole criterion for prioritizing the planning of health care delivery [84].

## Conclusion

In this paper, we explored the geospatial pattern of comorbidities prevalence among people with OA in Alberta, Canada. Our results highlighted the rural-urban disparities in the prevalence of most comorbidities and a distinct pattern in the prevalence of depression in major urban areas. These geographic differences help to explain the spatially varying health care needs and outcomes across the province, emphasize the need of health promotion with respect to OA and suggest where prevention programs can be targeted to those in need.

## Supplementary information

**Additional file 1: Appendix 1.** Case definitions for eight comorbid conditions. **Appendix 2.** Size of LGAs by rural-urban continuum. **Appendix 3.** Hot spots for each comorbidity group (queen contiguity with at least 8 neighbors). The authors created these maps using ArcMap 10.8 through the University of Calgary Educational Site License.

## Data Availability

The data that support the findings of this study are available from Alberta Health Services, but restrictions apply to the availability of these data, which were used under license for the current study, and so are not publicly available. Data are however available from the authors upon reasonable request and with permission of Alberta Health Services.

## Abbreviations

OA: Osteoarthritis
LGA: Local Geographic Area
COPD: Chronic Obstructive Pulmonary Disease
AHS: Alberta Health Services
AH: Alberta Health
AHCIP: Alberta Health Care Insurance Plan Population registry
DAD: Discharge Abstract Database
ACCS: Ambulatory Care Classification System
NACRS: National Ambulatory Care Reporting System
ICD: International Classification of Diseases
CI: Confidence Interval
SRR: Standardized Rates Ratio
HH: High-High cluster
LL: Low-Low cluster
HL: High-Low outlier
LH: Low-High outlier
