# Supplementary material for: Geospatial patterns of comorbidity prevalence among people with osteoarthritis in Alberta Canada
Source: BMC Public Health. 2020 Oct 15;20:1551. doi: 10.1186/s12889-020-09599-0 (PMC7559790; doi:10.1186/s12889-020-09599-0)

Appendix 1 Case definitions for eight comorbid conditions

|  | **ICD9-CM Diagnosis Codes Included** | **ICD10-CA Diagnosis Codes Included** | **Exclusions** | **Algorithm** | **References** |
| --- | --- | --- | --- | --- | --- |
| **Myocardial Infarction** | 410 | I21, I22 | N/A | 1 hospitalization for MI, with diagnoses codes in primary or secondary diagnosis fields. | McCormick et al., 2014^24^ Quan et al., 2005^25^ |
| **Cerebrovascular Disease** | 3623,43301,43311,43321,43331,43381,43391,43401,43411,43491, 436,430,431,435 | H341, I63, I64, I61, I60, G450, G451, G452, G453, G458, G459 | 43300, 43310, 43320, 43330, 43380, 43390, 43400, 43410, 43490, 437, 438, I65, I66, I67, I69, G45.4 | 1 hospital discharge (most responsible diagnosis) for different stroke conditions | Kokotailo & Hill, 2005 McCormick et al., 2015^26^ |
| **Congestive Heart Failure** | 39891,40201,40211,40291,40401,40403,40411,40413,40491,40493,4254,4255,4257,4258,4259,428 | I43,I50,I099,I110,I130,I132,I255,I420,I425,I426,I427,I428,I429,P290 |  | 1 hospitalization or physician claim in diagnosis field | Schultz et al., 2013  McCormick et al., 2014^27^  Quan et al., 2005^25^  Lee et al., 2005 |
| **Peripheral Vascular Disease** | 0930,4373,440,441,4431,4432,4438,4439,4471,5571,5579,V434,443 | I70,I71, I731,I738,I739,I771,I790,I792,K551,K558,K559,Z958,Z959 | N/A | 1 hospitalization or physician claim in diagnosis field | Fan et al., 2013^28^ |
| **Chronic Obstructive Pulmonary Disease** | 4168,4169,490,491,492,493,494,495,496,500,501,502,503,504,505,5064,5081,5088 | J40,J41,J42,J43,J44,J45,J46,J47,J60,J61,J62,J63,J64,J65,J66,J67,I278,I279,J684,J701,J703 | N/A | 1 hospitalization or physician claim in diagnosis field | Smidth et al. 2012^29^  Quan et al., 2005^25^ |
| **Depression** | 2962,2963,2965,3004,309,311 | F204,F313,F314,F315,F32,F33,F341,F412,F432 | N/A | 1 hospitalization or physician claim in diagnosis field | Townsend et al., 2012^30^  Lix et al., 2006  Martens et al., 2010 |
| **Diabetes** | 250 | E10, E11, E12, E13, E14 | Physician claims with procedure code having a prefix of “X” (radiology/xray), “E” (labs) or “B” (dentist, ophthalmologist, chiropractic visits) | 1 hospitalization for diabetes OR 2 physician claims in a 2-year period | Chen et al., 2010^31^ Leong et al., 2013^32^  Hux et al., 2002 |
| **Hypertension** | 401, 402, 403, 404, 405 | I10, I11, I12, I13, I15 | Physician claims with procedure code having a prefix of “X” (radiology/xray), “E” (labs) or “B” (dentist, ophthalmologist, chiropractic visits) | 1 hospitalization for hypertension OR 2 physician claims in a 2-year period | Quan et al., 2009^33^ |

Appendix 2 Size of LGAs by rural-urban continuum

| **Rural-Urban** | **NO. Of LGAs** | **Min (KM^2^)** | **Average (KM^2^)** | **Max (KM^2^)** |
| --- | --- | --- | --- | --- |
| **Metro** | 31 | 7 | 50 | 148 |
| **Moderate Metro** | 16 | 35 | 879 | 4,723 |
| **Urban** | 9 | 12 | 52 | 120 |
| **Moderate Urban** | 5 | 117 | 5,311 | 13,511 |
| **Rural Centre** | 6 | 1,649 | 5,370 | 9,115 |
| **Rural** | 53 | 880 | 5,036 | 22,563 |
| **Rural Remote** | 12 | 2,281 | 26,742 | 99,994 |
| **Alberta** | **132** | **7** | **5,020** | **99,994** |

Appendix 3: Hot spots for each comorbidity group (queen contiguity with at least 8 neighbors). The authors created these maps using ArcMap 10.8 through the University of Calgary Educational Site License


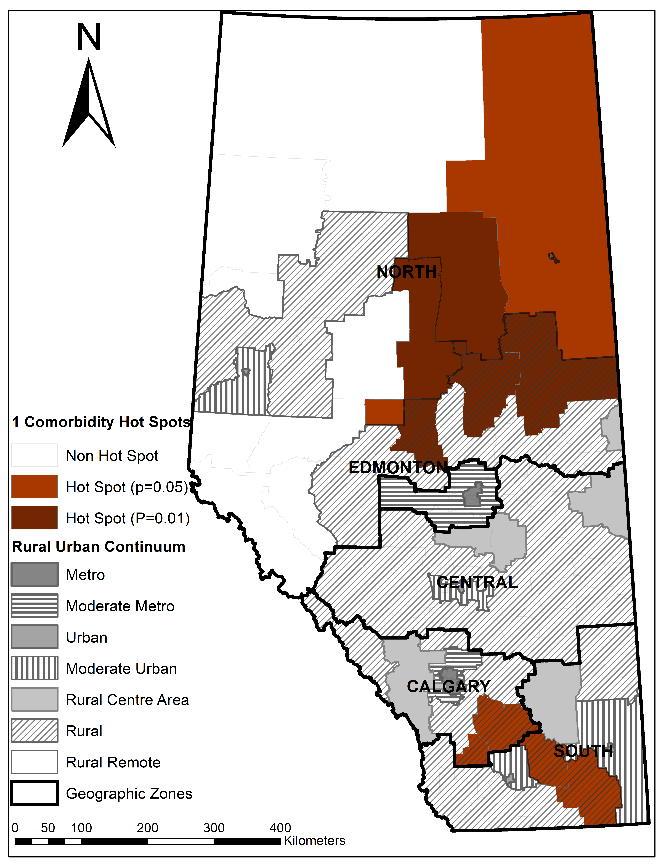

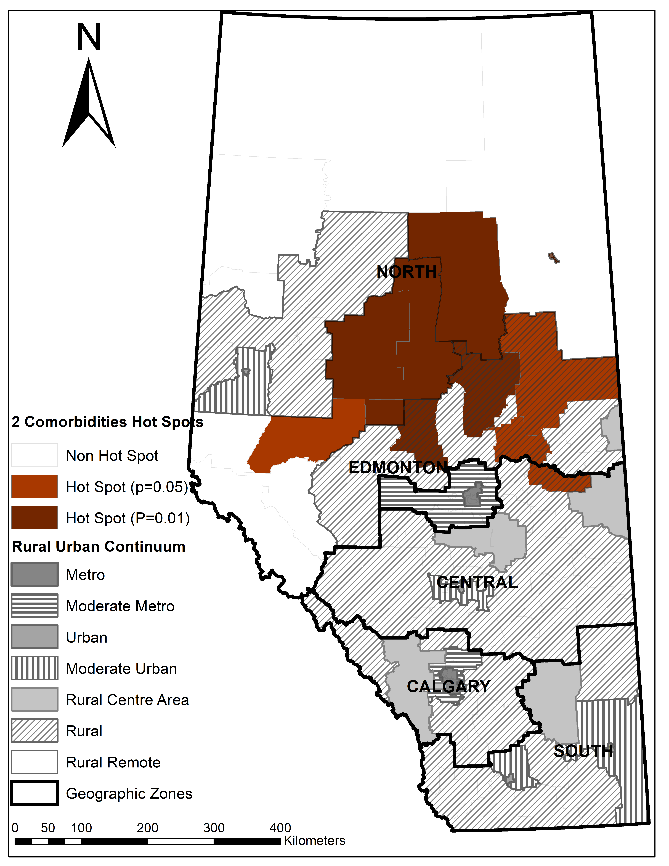

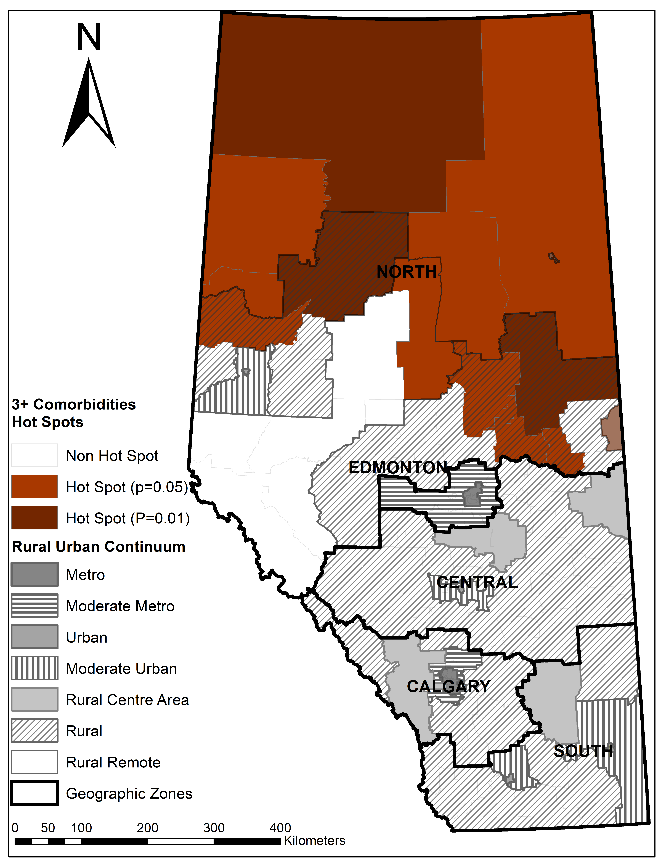

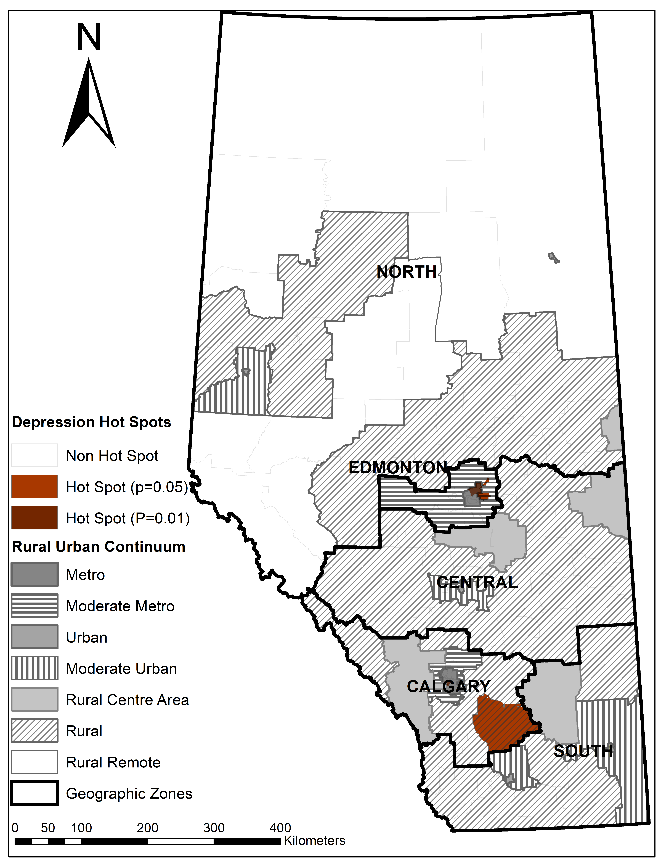

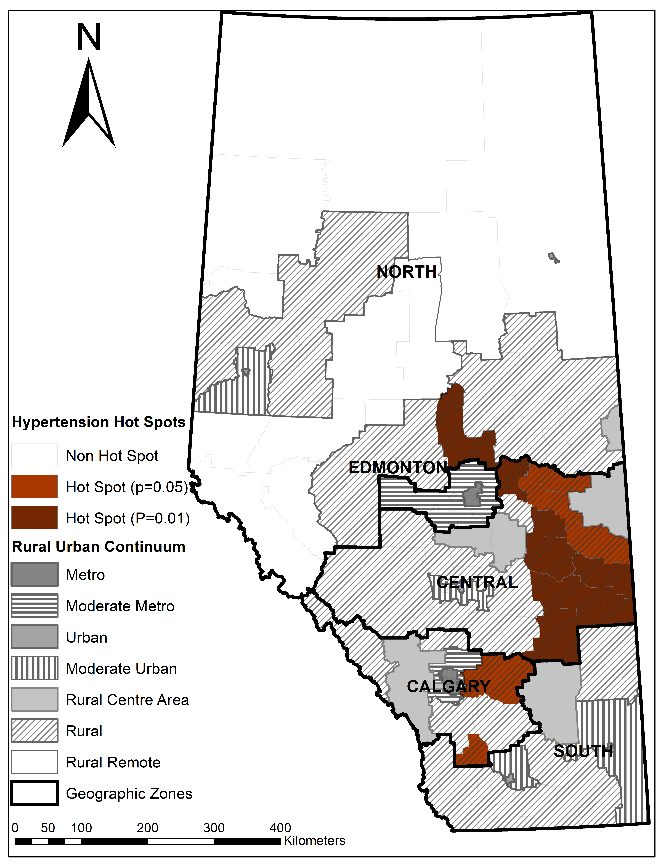

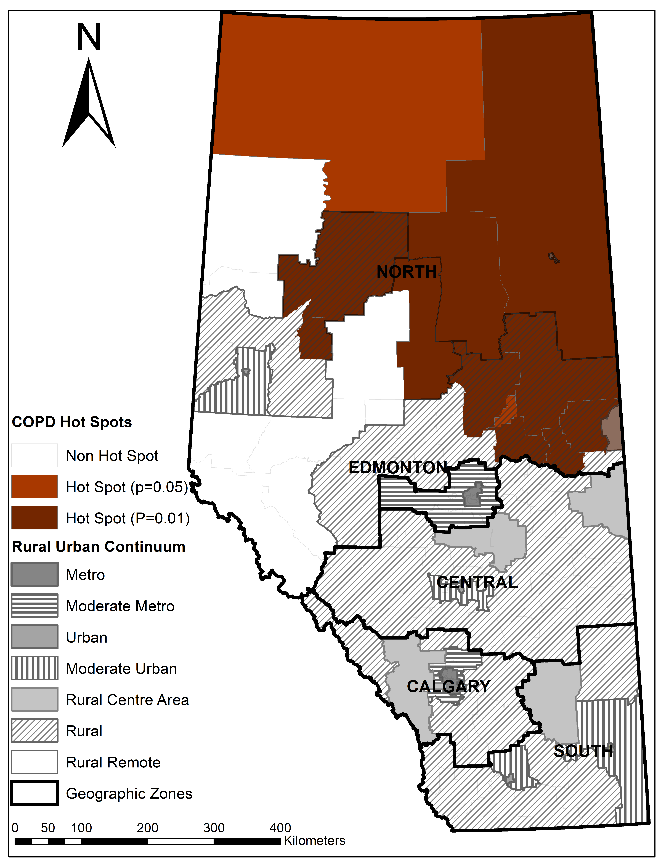

Supplement: Supplementary file 1 — Additional file 1: Appendix 1. Case definitions for eight comorbid conditions. Appendix 2. Size of LGAs by rural-urban continuum. Appendix 3. Hot spots for each comorbidity group (queen contiguity with at least 8 neighbors). The authors created these maps using ArcMap 10.8 through the University of Calgary Educational Site License. [file 12889_2020_9599_MOESM1_ESM.docx]
